# The Immunome in Two Inherited Forms of Pulmonary Fibrosis

**DOI:** 10.3389/fimmu.2018.00076

**Published:** 2018-01-31

**Authors:** Souheil El-Chemaly, Foo Cheung, Yuri Kotliarov, Kevin J. O’Brien, William A. Gahl, Jinguo Chen, Shira Y. Perl, Angélique Biancotto, Bernadette R. Gochuico

**Affiliations:** ^1^Division of Pulmonary and Critical Care Medicine, Brigham and Women’s Hospital, Boston, MA, United States; ^2^Trans-NIH Center for Human Immunology, Autoimmunity, and Inflammation (CHI), National Institutes of Health, Bethesda, MD, United States; ^3^Office of the Clinical Director, National Human Genome Research Institute, National Institutes of Health, Bethesda, MD, United States; ^4^Medical Genetics Branch, National Human Genome Research Institute, National Institutes of Health, Bethesda, MD, United States

**Keywords:** B-cell, cytokine, Hermansky–Pudlak syndrome, immunome, lymphocyte, pulmonary fibrosis, T-cell, telomere disease

## Abstract

The immunome (immune cell phenotype, gene expression, and serum cytokines profiling) in pulmonary fibrosis is incompletely defined. Studies focusing on inherited forms of pulmonary fibrosis provide insights into mechanisms of fibrotic lung disease in general. To define the cellular and molecular immunologic phenotype in peripheral blood, high-dimensional flow cytometry and large-scale gene expression of peripheral blood mononuclear cells and serum proteomic multiplex analyses were performed and compared in a cohort with familial pulmonary fibrosis (FPF), an autosomal dominant disorder with incomplete penetrance; Hermansky–Pudlak syndrome pulmonary fibrosis (HPSPF), a rare autosomal recessive disorder; and their unaffected relatives. Our results showed high peripheral blood concentrations of activated central memory helper cells in patients with FPF. Proportions of CD38^+^ memory CD27^−^ B-cells, IgA^+^ memory CD27^+^ B-cells, IgM^+^ and IgD^+^ B-cells, and CD39^+^ T helper cells were increased whereas those of CD39^−^ T helper cells were reduced in patients affected with either familial or HPSPF. Gene expression and serum proteomic analyses revealed enrichment of upregulated genes associated with mitosis and cell cycle control in circulating mononuclear cells as well as altered levels of several analytes, including leptin, cytokines, and growth factors. In conclusion, dysregulation of the extra-pulmonary immunome is a phenotypic feature of FPF or HPSPF. Further studies investigating the blood immunome are indicated to determine the role of immune system dysregulation in the pathogenesis of pulmonary fibrosis.

**Clinical Trial Registration:**

www.ClinicalTrials.gov, identifiers NCT00968084, NCT01200823, NCT00001456, and NCT00084305.

## Introduction

Pulmonary fibrosis, a chronic progressive interstitial lung disease of unknown etiology, is associated with a poor prognosis and often culminates in lung transplantation or death from respiratory failure (1). Idiopathic pulmonary fibrosis is a common, genetically heterogeneous fibrotic lung disease that typically affects older individuals without known predisposing factors (2). Insights into the pathogenesis of pulmonary fibrosis may be gleaned from studies focusing on inherited monogenic or oligogenic disorders associated with highly penetrant fibrotic lung disease in younger subpopulations. Familial pulmonary fibrosis (FPF) and Hermansky–Pudlak syndrome (HPS) are two genetic disorders that may serve as models for studying pulmonary fibrosis.

Familial pulmonary fibrosis, defined as the development of pulmonary fibrosis of unknown etiology in two or more first-degree relatives, comprises less than 5% of idiopathic pulmonary fibrosis patients and exhibits an autosomal dominant inheritance with incomplete penetrance (3). Patients with FPF generally manifest with symptoms in the sixth decade of life, and progressive preclinical interstitial lung disease can be detected approximately one to two decades before the onset of symptomatic disease (4–6). The etiology of FPF is unclear, but a subpopulation with telomere disease or dyskeratosis congenita have short telomeres associated with deficient telomerase complex and monoallelic mutations in *TERT, TERC, RTEL1, PARN, DKC1*, or *TINF2* (5, 7–11). There are FPF kindreds without telomere-related disorders, including those with mutations in genes encoding surfactant proteins (i.e., *SFTPC* and *SFTPA2*) and others with unidentified mutations (12, 13).

Hermansky–Pudlak syndrome is a rare multisystem autosomal recessive disorder with abnormal biogenesis of lysosome-related organelles (14, 15). Ten genetic types have been reported, and each type is associated with a deficiency in the Adaptor Protein-3 complex or the Biogenesis of Lysosome-related Organelles Complex (BLOC)-1, BLOC-2, or BLOC-3 (15–17). Clinical manifestations observed in patients with various HPS types include oculocutaneous albinism, bleeding tendency due to a platelet storage pool defect, and colitis (14). HPS type-specific manifestations include neutropenia, immunodeficiency, and pulmonary fibrosis (18–20). Progressive pulmonary fibrosis develops in middle-aged adults with HPS-1 and HPS-4, which are BLOC-3 disorders, as well as children and young adults with HPS-2, which is due to Adaptor Protein-3 complex deficiency (19, 21, 22). HPS-1, the most common HPS type associated with fibrotic lung disease, usually affects individuals of Puerto Rican ethnicity, who harbor a mutation (c.1472_1487dup16) in *HPS1* due to a genetic founder effect (14).

Phenotypic alterations in lung inflammatory cells are found in the alveolar milieu in pulmonary fibrosis and may contribute to the pathogenesis of disease. For example, CD11b^+^ alveolar macrophages isolated from patients with idiopathic pulmonary fibrosis, but not healthy volunteers, can transdifferentiate to lymphatic endothelial cells *in vitro* (23). In addition, alveolar lymphocytosis with activated CD4^+^ cells is a feature of preclinical FPF, and alveolar inflammation precedes the development of fibrotic lung disease by decades in patients with a telomerase mutation (4, 6). High-resolution computed tomography (HRCT) scans from patients with HPS-1 lung disease show ground glass opacifications, which may indicate alveolar inflammation (24, 25). Consistent with these findings, HPS-1 is associated with high concentrations of bronchoalveolar lavage cells, alveolar macrophage activation, and fibrogenic mast cells (26, 27). These data indicate that immune cell dysfunction and influx into lung tissue are features of patients at high risk of inherited pulmonary fibrosis and may lead to alveolar injury and the development of early lung fibrosis.

Given these findings of dysregulated immune cells in the lungs of patients with FPF and HPS-1 pulmonary fibrosis, we investigated the immunome and immune cells in peripheral blood from patients with these inherited fibrotic lung diseases. We report high concentrations of activated T-cell and B-cell subpopulations associated with increased cytokine levels in this cohort. Consistent with these findings, leptin concentrations were high, and expression of genes involved with mitosis and cell replication in peripheral blood mononuclear cells (PBMCs) was upregulated and enriched.

## Materials and Methods

### Subject Consent and Inclusion Criteria

Written informed consent in accordance with the Declaration of Helsinki was obtained from all subjects, who enrolled in protocols 09-H-0201 (Clinical Trials number, NCT00968084; Screening Protocol for Subjects Being Evaluated for Center for Human Immunology, Autoimmunity, and Inflammatory Diseases (CHI) Protocols), 10-H-0162 (Clinical Trials number, NCT01200823; Collection of Blood, Bone Marrow, Leukapheresis, and Tissue Biopsy Samples from Patients and their Family members for Center for Human Immunology, Autoimmunity, and Inflammatory Diseases (CHI) Laboratory Research Studies), 95-HG-0193 (Clinical Trials number, NCT00001456; Clinical and Basic Investigations into HPS), and/or 04-HG-0211 (Clinical Trials number, NCT00084305; Procurement and Analysis of Specimens from Individuals with Pulmonary Fibrosis), which were approved by the Institutional Review Board of the National Heart, Lung and Blood Institute or the National Human Genome Research Institute. Patients with HPS were diagnosed based on absent platelet delta granules on whole mount electron microscopy analysis and genotyping as described (28); pulmonary fibrosis was diagnosed in patients with HPS by HRCT scan (22). Patients with FPF were diagnosed as described (14). Unaffected first-degree relatives of patients with pulmonary fibrosis had no radiographic evidence of pulmonary fibrosis, and genotyping showed that these individuals did not have the pathogenic mutations associated with disease in their relatives.

### Clinical Testing and Radiographic Imaging

Pulmonary function tests and prone HRCT scans of the chest without intravenous contrast were performed as described (29). Complete blood count testing with differential analysis was performed on peripheral blood specimens obtained by venopuncture. Clinical evaluations for all subjects were performed at the National Institutes of Health Clinical Center in Bethesda, MD, USA.

### Sample Processing

Blood specimens were collected in heparinized tubes and PBMCs were isolated using Leucosep tubes with Ficoll-Paque Plus density gradient media and centrifugation according to manufacturer and CHI standard operational procedures (CHI-SOP; http://www.nhlbi.nih.gov/resources/chi/documents/SOP). Fresh isolated PBMCs for RNA isolation were directly lysed in 700 µl of QIAzol (QIAGEN, Hilden, Germany) and stored at −80°C until isolation. Human PBMCs used for deep phenotyping by 15 color flow cytometry analysis were cryopreserved in freezing medium consisting of 10% dimethylsulfoxide and 90% heat inactivated fetal bovine serum. Cells suspended in freezing medium were cryopreserved using Planer 750Plus™ Controlled Rate Freezer (Planer PLC, Middlesex, UK) at control-rate steps to a temperature of −120°C to minimize cell damage and were then transferred into the vapor phase of a liquid nitrogen tank until use. Serums were collected using 8 ml SST tubes (Becton Dickinson, San Jose, CA, USA) according to CHI-SOPs.

### RNA Isolation and Microarray Hybridization

Total RNA was isolated from 1e7 freshly isolated PBMCs using miRNeasy kit (QIAGEN) according to the manufacturer’s instructions and eluted in 40 µl of elution buffer with the addition of 1 µl of RNase inhibitor (Life Sciences, Carlsbad, CA, USA). RNA quality was assessed by Agilent Bioanalyzer and quantified by NanoDrop 2000 (ThermoFisher Scientific, Waltham, MA, USA). 300 ng of total RNA were amplified using Ambion WT expression kit (Life Sciences) according to the manufacturer’s instructions. Fragmented single-stranded sense cDNA were terminally labeled and hybridized to Human 1.0 ST GeneChip arrays (Affymetrix, Santa Clara, CA, USA) and stained on a GeneChip Fluidics Station 450 (Affymetrix), according to the respective manufacturers’ instructions. Arrays were scanned on a GeneChip Scanner 3000 7G (Affymetrix).

### Flow Cytometric Immunophenotyping

Immunome profiling was performed using frozen samples within a single analytic run for each assay, thus minimizing batch effects. Once thawed, cells were washed in FACS buffer (1× phosphate-buffered saline, 0.5% fetal calf serum, 0.5% normal mouse serum, and 0.02% NaN_3_) and were stained with LIVE/DEAD Aqua fixable viability dye (Life Technologies, Carlsbad, CA, USA), washed two times with FACS buffer. Then, cells were incubated with fluorochrome-conjugated antibodies from our published panel for surface staining (30, 31) for T lineage, B lineage, and NK lineage. After incubation with antibodies for 30 min, cells washed two times with FACS buffer, and fixed in 1% paraformaldehyde. Cell were acquired on a Fortessa flow cytometer equipped with 405, 488, 532, and 638 laser lines using DIVA™ 6.1.2 software (Becton Dickinson). Data were analyzed with FlowJo™ software version 9.7.6 (Tree Star, San Carlos, CA, USA). All populations are expressed as the percent of parent gate.

### Cytokine Measurement

Serum samples were analyzed using five different Luminex cytokine kits (Bio-Rad, Hercules, CA, USA) (Table S1 in Supplementary Material). All assays were performed according to the instructions provided by the manufacturer. Briefly, median fluorescence intensities were collected on a Luminex-200 instrument (Bio-Rad), using Bio-Plex Manager software version 6.2 (Bio-Rad). Standard curves for each cytokine were generated using the premixed lyophilized standards provided in the kits, and concentrations were determined from the standard curve using a 5-point regression to transform the median fluorescence intensity values into concentrations. Each sample was run in duplicate and the average of the duplicates was used as the measured concentration. All analytes were detectable in at least one patient, except GM-CSF, IL-3, and LIF. Any value that was below detection level was replaced by the limit of detection divided by 2. Analyses were performed using Data Pro Manager 1.02 (Bio-Rad).

### Data Analysis

For age and pulmonary function tests, values are expressed as mean ± SEM; significance of differences between groups was analyzed using unpaired Student’s *t*-test. For complete blood count, flow cytometry and Luminex datasets, statistics, plotting, and quality control were conducted by interactive tools developed internally using Shiny (RStudio, Boston, MA, USA) (Foo Cheung); significance of differences between groups was evaluated using Mann–Whitney *U* test. *p*-Values of ≤0.1 were considered significant; *p*-values were adjusted for multiple testing using Benjamini and Hochberg’s false discovery rate (FDR) with Bonferroni corrections.

Quality control and primary data analysis for the Affymetrix mRNA microarray experiment were performed as described (32). Briefly, Affymetrix CEL files were processed with Affymetrix Power Tools for probeset summarization, normalization and log_2_-transformation (RMA with sketch quantile normalization). Quality assessment with arrayQualityMetrics R package detected no outlying arrays. Probesets (1) not annotated with any gene symbol, (2) associated with multiple gene symbols, and (3) having very low variability (with interquantile range across all samples less than 0.15) were removed. When multiple probesets were associated with the same gene, the probeset with the highest correlation to the first principal component computed from intensities of these probesets in many previously collected arrays was selected. After filtering, 15,024 genes remained. Microarray data were submitted to Gene Expression Omnibus and are publicly available (GEO accession number GSE107797). Linear models applying R/Bioconductor *limma* package with contrasts to specify pairwise comparisons were used to determine genes differentially expressed between patient groups. *p*-Values were adjusted for multiple testing using Benjamini and Hochberg’s FDR method. Genes were identified as differentially expressed with FDR-adjusted *p*-values less than 0.1 and absolute log_2_-fold change larger than 0.3 in at least for one comparison. Blood transcription modules enrichment analysis (33) was performed on limma contrasts using tmod R package (34). The genes were ordered by minimum significant difference that uses both effect size and statistical significance.

## Results

### Subject Characteristics

Twelve patients with HPS pulmonary fibrosis (2 males and 10 females), 5 with FPF associated with telomere disease (1 male and 4 females), and 4 unaffected relatives (URels) (3 males and 1 female) were studied (Table 1). Patients were not affected with acute infection or under treatment with immunomodulatory drugs during their evaluations when samples were obtained. FPF probands included two siblings and three unrelated individuals; these probands were significantly older than patients with HPS pulmonary fibrosis or URels. Eleven of 12 patients with HPS had homozygous mutations (c.1472_1487dup16) in *HPS1* (Table 2); one patient with HPS had a compound heterozygous mutation in *HPS1*. All patients with FPF had telomere disease; four patients had single mutations in *TERT*, and one had a variant in *TERC*.

**Table 1:** Patient characteristics.

	FPF	HPSPF	URel	*p*-Value
Age (years)	63.4 ± 3.2	42.0 ± 2.9	43.3 ± 9.3	<0.001^a^
				0.060^b^
				0.86^c^
Gender (M/F)	1/4	2/10	3/1	
FVC (% predicted)	89.0 ± 10.5	67.8 ± 5.7	120.5 ± 3.6	0.073^a^
				0.037^b^
				<0.001^c^
TLC (% predicted)	87.0 ± 7.6	73.6 ± 6.1	115.3 ± 5.0	0.23^a^
				0.022^b^
				0.002^c^
DLCOa (% predicted)	55.4 ± 11.8	43.2 ± 4.6	92.5 ± 8.2	0.25^a^
				0.045^b^
				<0.001^c^
HRCT scan	Fibrosis	Fibrosis	No fibrosis	

*FPF, familial pulmonary fibrosis; HPSPF, Hermansky–Pudlak syndrome pulmonary fibrosis; URel, unaffected relative; M, male; F, female; FVC, forced vital capacity; TLC, total lung capacity; DLCOa, diffusion capacity (adjusted); HRCT, high-resolution computed tomography*.

*^a^FPF vs HPSPF*.

*^b^FPF vs URel*.

*^c^HPSPF vs URel*.

**Table 2:** Genetic mutations.

Patient	Group	Allele 1 mutation	Allele 2 mutation
1	URel		
2	FPF	*TERT* (870 Phe>LLe)	
3	FPF	*TERT* (c.3251 G>C; R1084P)	
4	HPSPF	*HPS1* (c.1472_1487dup16)	*HPS1* (c.1472_1487dup16)
5	HPSPF	*HPS1* (c.1472_1487dup16)	*HPS1* (c.1472_1487dup16)
6	FPF	*TERT* (c.3251 G>C; R1084P)	
7	HPSPF	*HPS1* (c.1472_1487dup16)	*HPS1* (c.1472_1487dup16)
8	HPSPF	*HPS1* (c.1472_1487dup16)	*HPS1* (c.1472_1487dup16)
9	HPSPF	*HPS1* (c.1472_1487dup16)	*HPS1* (c.1472_1487dup16)
10	URel		
11	FPF	*TERT* (P59S)	
12	HPSPF	*HPS1* (c.1472_1487dup16)	*HPS1* (c.1472_1487dup16)
13	FPF	*TERC* (r.119 C>G)	
14	HPSPF	*HPS1* (c.1472_1487dup16)	*HPS1* (c.937G>A; p.Gly313Ser)
15	URel		
16	URel		
17	HPSPF	*HPS1* (c.1472_1487dup16)	*HPS1* (c.1472_1487dup16)
18	HPSPF	*HPS1* (c.1472_1487dup16)	*HPS1* (c.1472_1487dup16)
19	HPSPF	*HPS1* (c.1472_1487dup16)	*HPS1* (c.1472_1487dup16)
20	HPSPF	*HPS1* (c.1472_1487dup16)	*HPS1* (c.1472_1487dup16)
21	HPSPF	*HPS1* (c.1472_1487dup16)	*HPS1* (c.1472_1487dup16)

*FPF, familial pulmonary fibrosis; HPSPF, Hermansky–Pudlak syndrome pulmonary fibrosis; URel, unaffected relative*.

**Figure 1:**
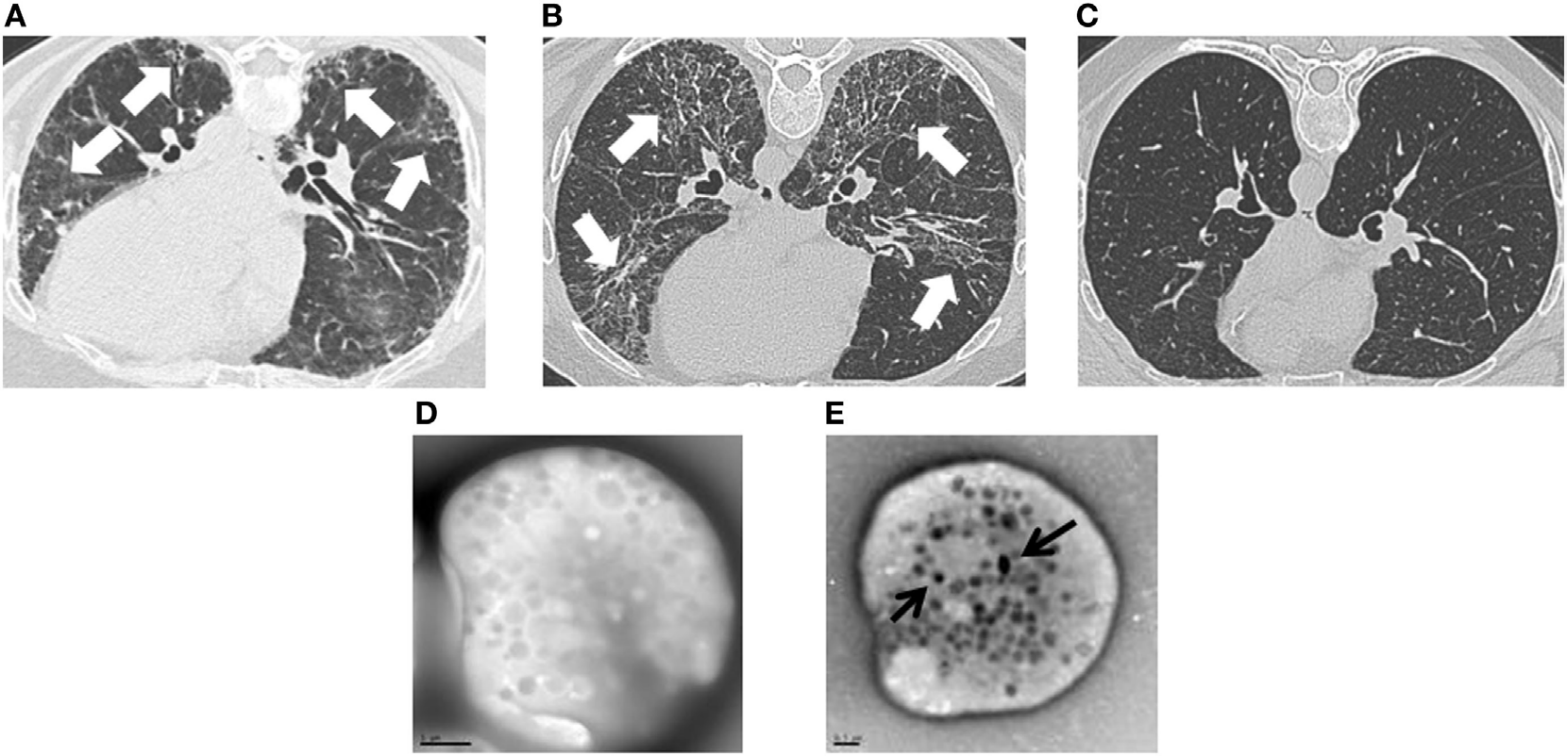
Clinical images in patients with familial pulmonary fibrosis (FPF), Hermansky–Pudlak syndrome pulmonary fibrosis (HPSPF), and unaffected relatives (URels). High-resolution computed tomography scan images demonstrate lung fibrosis (white arrows) in patients with FPF **(A)** or HPSPF **(B)**, and not in an URel **(C)**. Whole mount electron microscopy images show absent platelet delta granules in a patient with Hermansky–Pudlak syndrome **(D)**, size bar = 1 μm; normal control platelets contain delta granules (black arrows) **(E)**, size bar = 0.5 μm.

Clinical testing included lung function measurements and imaging studies. Forced vital capacity (FVC), total lung capacity (TLC), and diffusion capacity were significantly lower in patients with HPS pulmonary fibrosis or FPF compared with URels (Table 1). Although patients with HPS pulmonary fibrosis had significantly lower FVC than FPF probands, TLC, and diffusion capacity were not significantly different between these two groups. HRCT scans showed bilateral parenchymal fibrotic lung disease in patients with FPF or HPS pulmonary fibrosis, and not URels (Figures 1A–C). Whole mount electron microscopy of platelets from patients with HPS demonstrated absence of delta granules, which are typically found in normal platelets (Figures 1D,E).

### Peripheral Blood Cell Phenotyping

**Figure 2:**
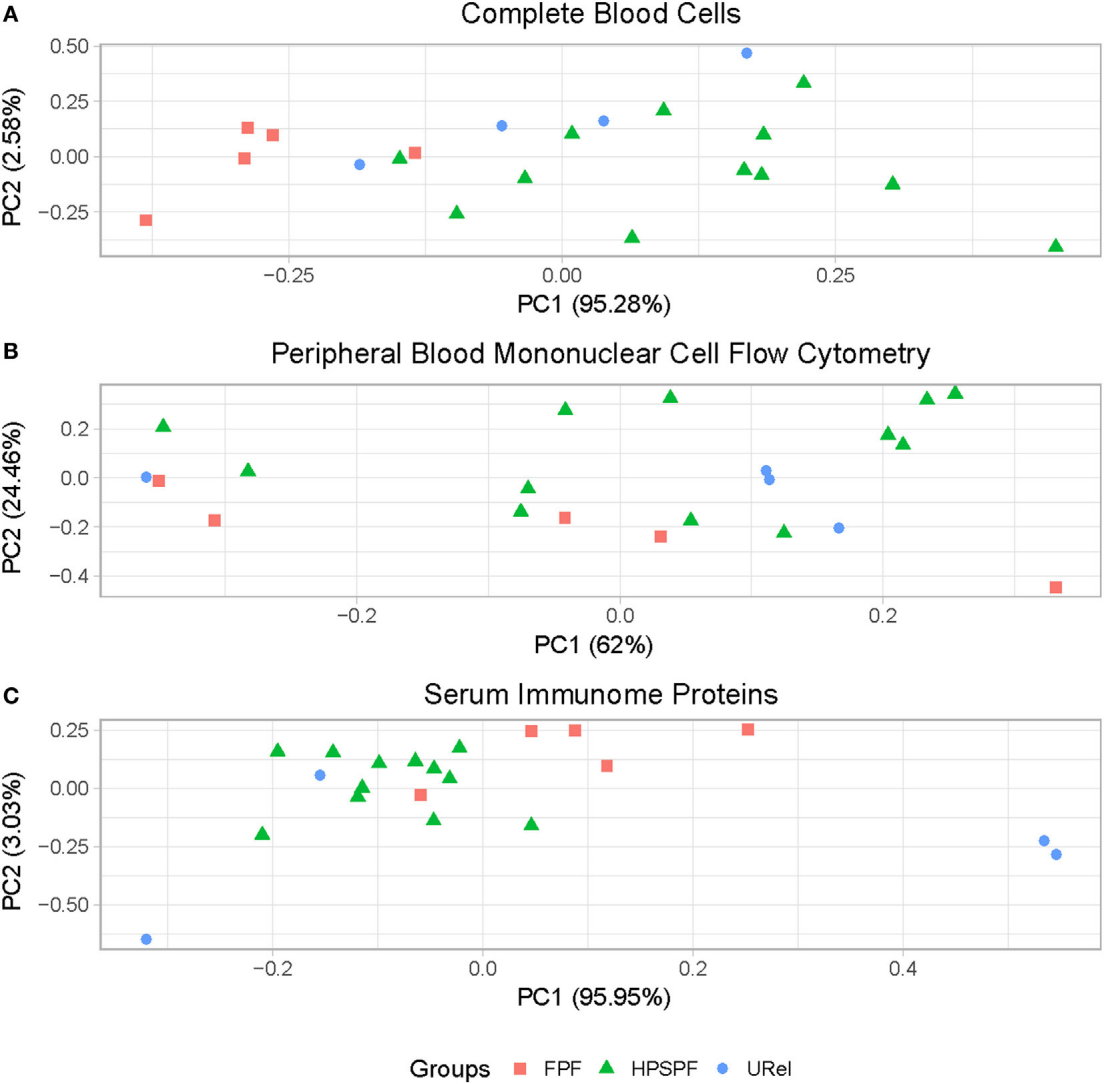
Principal component analysis (PCA) of peripheral blood cells and serum immunome proteins in patients with familial pulmonary fibrosis (FPF), Hermansky–Pudlak syndrome pulmonary fibrosis (HPSPF), and unaffected relatives (URels). PCA of complete blood cell parameters shows clustering of patients with FPF, HPSPF, and URel **(A)**. Subpopulations of patient groups are not found on PCA of peripheral blood mononuclear cell flow cytometry data **(B)**. Patient groups also cluster with PCA of serum immunome proteomic data; one patient with FPF and their unaffected sibling segregate together as outliers **(C)**.

**Figure 3:**
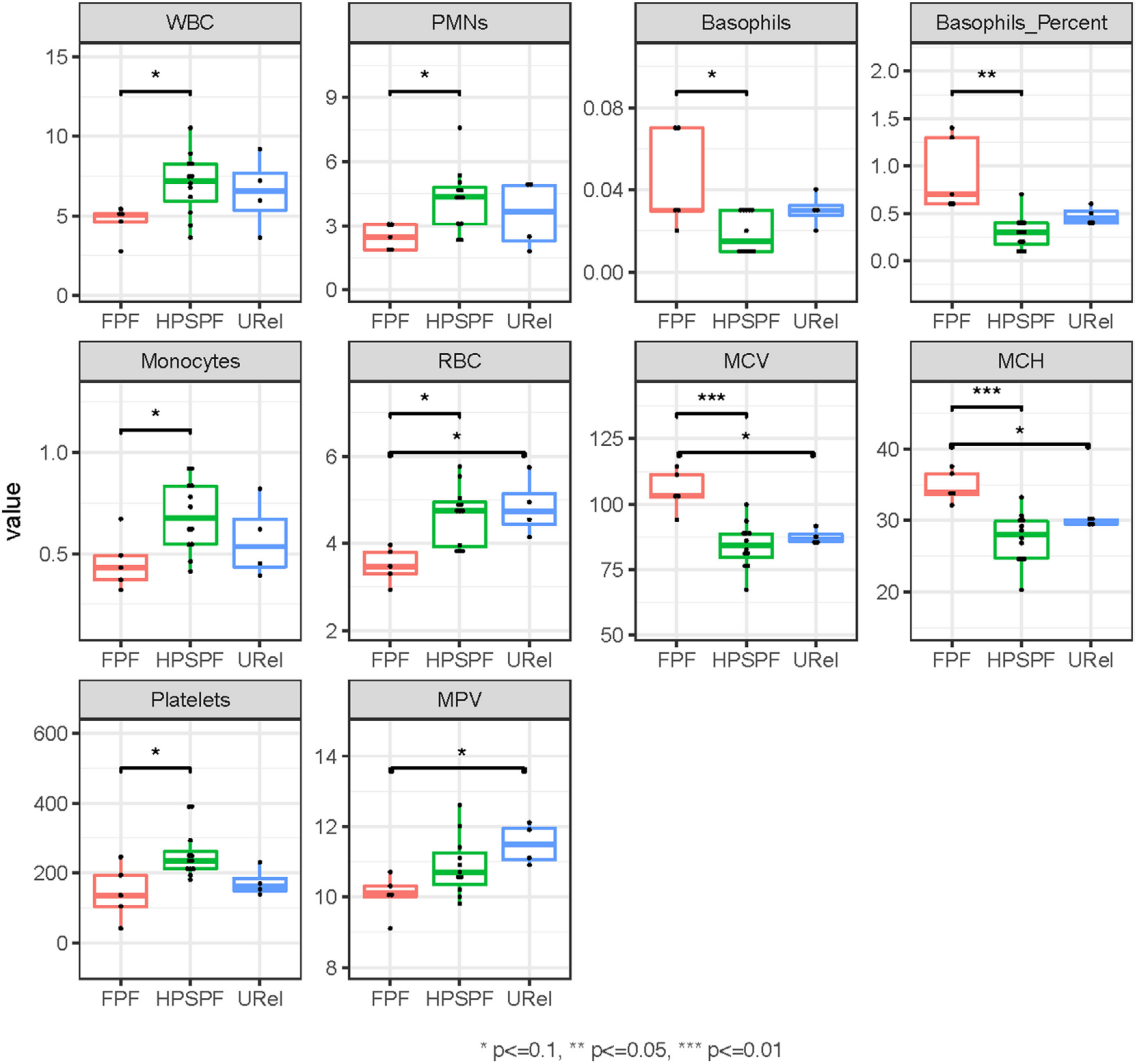
Phenotyping of peripheral blood cell parameters in patients with familial pulmonary fibrosis (FPF), Hermansky–Pudlak syndrome pulmonary fibrosis (HPSPF), and unaffected relatives (URels). Concentrations of white blood cell (WBC), polymorphonuclear leukocyte (PMNs), basophils, monocytes, and platelets as well as basophil percent were significantly different in patients with FPF compared with those with HPSPF. Red blood cell (RBC) counts, mean corpuscular volume (MCV), mean corpuscular hemoglobin (MCH), and mean platelet volume (MPV) were also significantly different between groups as shown.

Principal component analysis (PCA) of complete blood cell data demonstrated clustering of patient groups (Figure 2A). Basophil percentage as well as white blood cell (WBC), polymorphonuclear leukocyte, basophil, monocyte, and platelet concentrations were significantly different in patients with FPF compared with patients with HPS pulmonary fibrosis (Figure 3). Red blood cell (RBC) counts, mean corpuscular volume, and mean corpuscular hemoglobin were significantly different in patients with FPF compared with patients with HPS pulmonary fibrosis or URels, and mean platelet volume was significantly lower in patients with FPF compared with URels (Figure 3).

**Figure 4:**
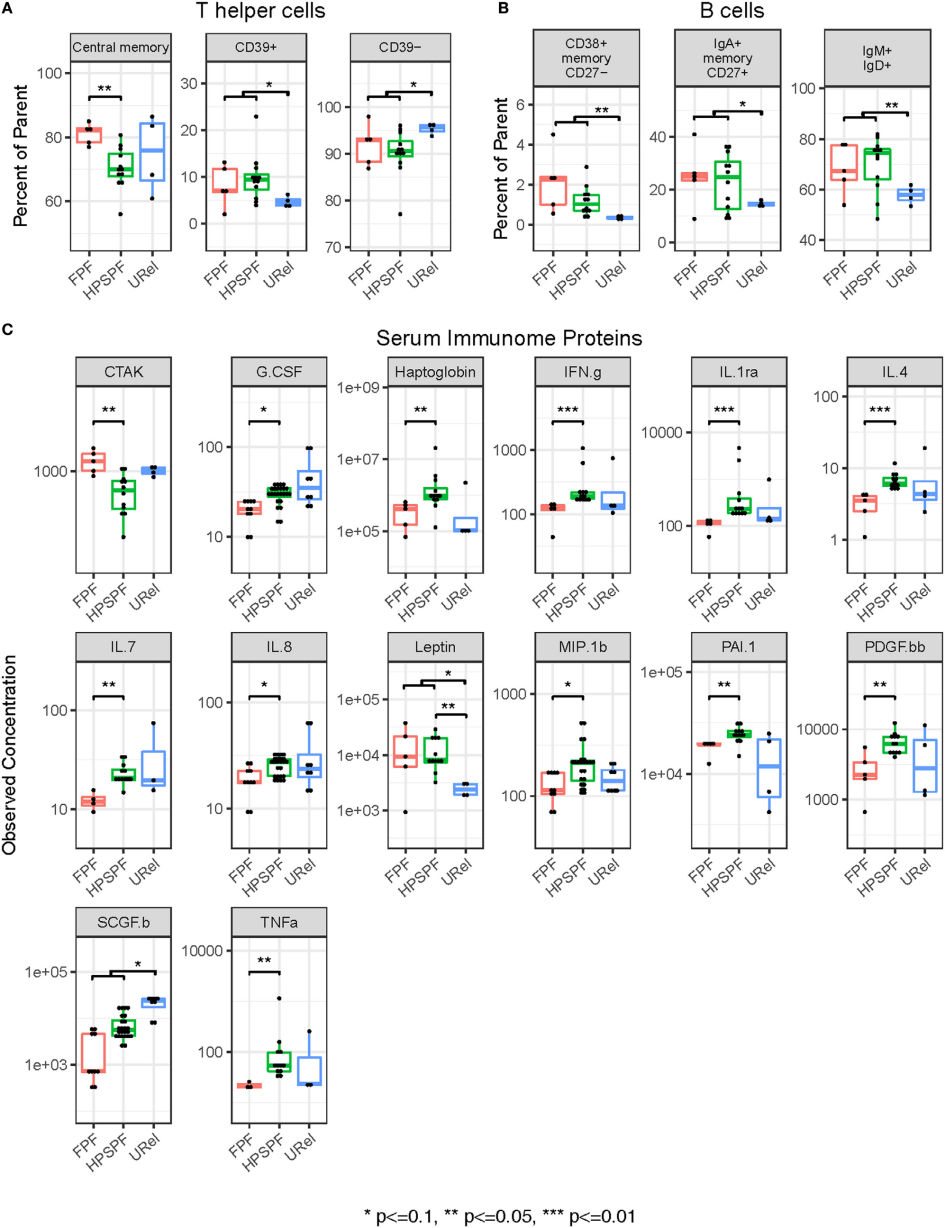
Flow cytometry of peripheral blood mononuclear cell populations and serum immunome profile in patients with familial pulmonary fibrosis (FPF), Hermansky–Pudlak syndrome pulmonary fibrosis (HPSPF), and unaffected relatives (URel). Percentages of central memory helper cells were significantly higher in patients with FPF compared with patients with HPSPF **(A)**. Percentages of CD38^+^ memory CD27^−^ B-cells, IgA^+^ memory CD27^+^ B-cells, IgM^+^ and IgD^+^ B-cells, CD39^+^ T helper cells, and CD39^−^ T helper cells were significantly different in patients affected with either FPF or HPSPF compared with URel **(A,B)**. Serum concentrations of chemokine ligand-27 (CTAK), granulocyte colony-stimulation factor (G.CSF), haptoglobin, interferon-γ (IFN.g), interleukin-1rα (IL.1ra), interleukin-4 (IL.4), interleukin-7 (IL.7), interleukin-8 (IL.8), leptin, macrophage inflammatory protein 1β (MIP.1b), plasminogen activator inhibitor-1 (PAI.1), platelet-derived growth factor-bb (PDGF.bb), stem cell growth factor-β (SCGF.b), and tumor necrosis factor-α (TNFa) were significantly different between groups as shown **(C)**.

Flow cytometry of PBMCs showed no significant differences between groups in percentages of CD4^+^ helper, CD8^+^ cytotoxic, CD45RO^+^ memory, or FOXP3^+^ regulatory T-cells or CD19^+^ B-cells (data not shown). However, significantly higher percentages of central memory helper cells were found in patients with FPF compared with those with HPS pulmonary fibrosis (Figure 4A). Percentages of CD38^+^ memory CD27^−^ B-cells, IgA^+^ memory CD27^+^ B-cells, IgM^+^ and IgD^+^ B-cells, and CD39^+^ T helper cells were significantly higher whereas CD39^−^ T helper cells were significantly lower in patients with either FPF or HPS pulmonary fibrosis compared with URels (Figures 4A,B). Despite these differences, subpopulations of patient groups were not found on PCA of PBMC flow cytometry data (Figure 2B).

### Blood Immunome Profile

Proteomic analysis of the serum immunome was performed in our cohort with inherited forms of pulmonary fibrosis to identify circulating cytokines, chemokines and growth factors associated with their peripheral blood immune cell phenotype. Serum concentrations of leptin were significantly higher in patients with HPS pulmonary fibrosis with or without patients with FPF compared with URels (Figure 4C). Levels of granulocyte colony-stimulation factor, haptoglobin, interferon-γ (IFN.g), interleukin-1rα, interleukin-4 (IL.4), interleukin-7, interleukin-8, macrophage inflammatory protein 1β (chemokine ligand-4), plasminogen activator inhibitor-1, platelet-derived growth factor-bb, and tumor necrosis factor-α were significantly higher in patients with HPS pulmonary fibrosis compared with those with FPF (Figure 4C). Concentrations of chemokine ligand-27 (CCL27, CTAK) were significantly higher in patients with FPF compared with patients with HPS pulmonary fibrosis, and concentrations of stem cell growth factor-β were significantly lower in patients with HPS pulmonary fibrosis or FPF compared with URels (Figure 4C). Some segregation of patient groups was evident on PCA of serum immunome proteomic data (Figure 2C); one patient with FPF and that same person’s unaffected sibling were outliers who segregated together.

**Figure 5:**
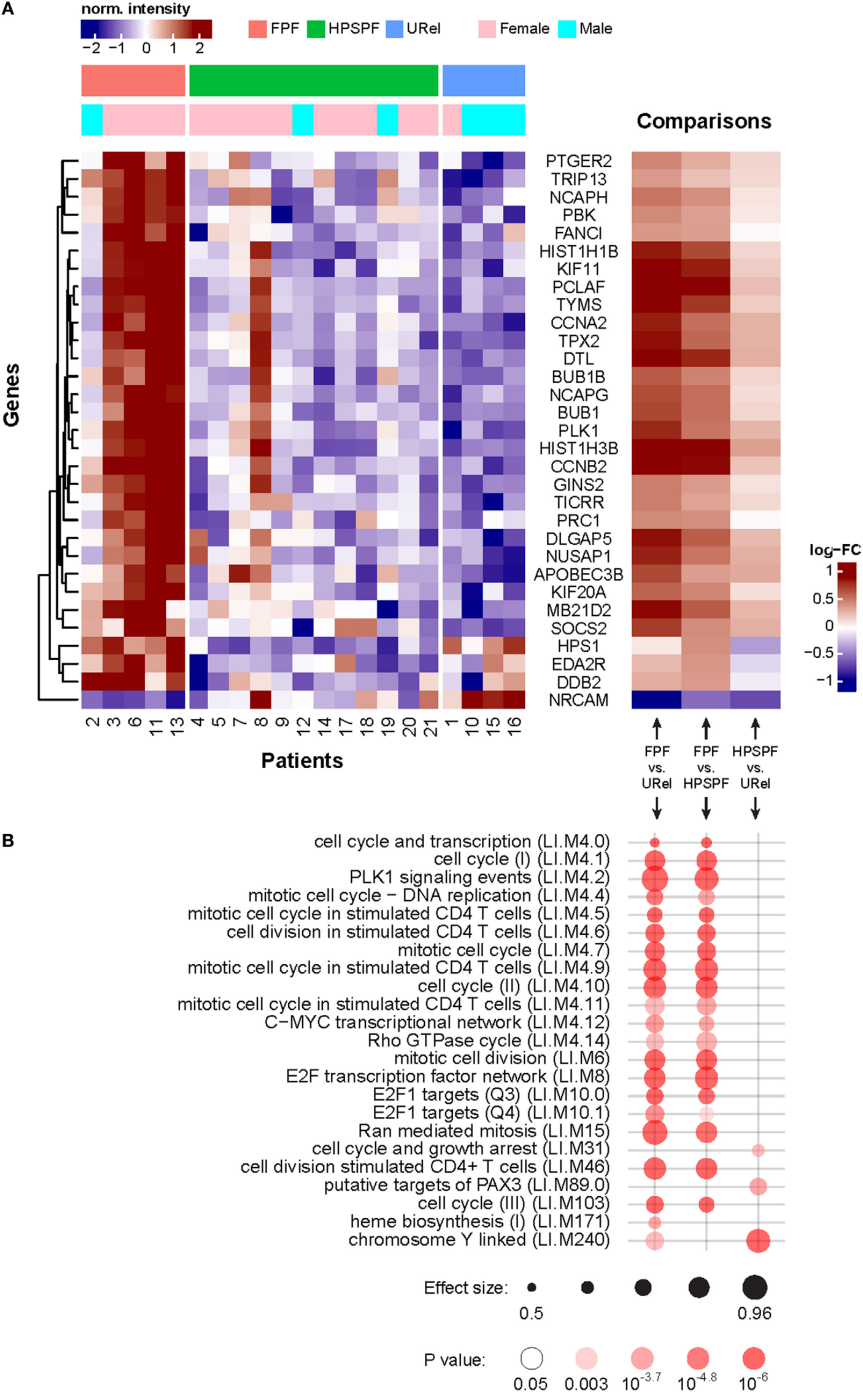
Analysis of peripheral blood mononuclear cell microarray expression data in patients with familial pulmonary fibrosis (FPF), Hermansky–Pudlak syndrome pulmonary fibrosis (HPSPF), and unaffected relatives (URel). Heatmaps of relative expression intensities of 31 differentially expressed genes with false discovery rate-adjusted *p*-value <0.1 and absolute log_2_-fold change > 0.3 in any pairwise comparison between three groups are displayed **(A)**. Significantly enriched blood transcription modules with adjusted *p*-value <10^−3^ in at least one of three pairwise comparisons are shown **(B)**.

Large-scale PBMC gene expression analysis was conducted to further investigate the blood immunome in these inherited disorders. Genes involved with mitosis and cell cycle regulation were enriched among the differentially expressed genes in patients with FPF (Figures 5A,B). Several genes encoding proteins involved with DNA biosynthesis or replication (e.g., *NCAPH, TYMS, CCNA2, PLK1, HIST1H1B, DTL, NCAPG, HIST1H3B, GINS2*, and *TICRR*), DNA repair (e.g., *TRIP13, FANCI, HIST1H1B, PCLAF, TYMS, DTL, HIST1H3B*, and *DDB2*), microtubule assembly or function (e.g., *KIF11, TPX2, BUB1B, BUB1, PLK1, PRC1, DLGAP5, NUSAP1*, and *KIF20A*), transcription regulation (e.g., *HIST1H1B* and *HIST1H3B*), or lymphoid activation (e.g., *PBK*) were upregulated in PBMCs from patients with FPF. A gene encoding a neuronal cell adhesion molecule (i.e., *NRCAM*) was the only one that was downregulated in PBMCs from patients with FPF (35). In these patients with HPS, gene expression was dissimilar in patient 8, who had atypical radiographic findings of predominantly focal lung disease with lobar pulmonary fibrosis and bronchiectasis, Notably, *HPS1* was downregulated in patients with HPS-1 pulmonary fibrosis compared with patients with FPF or URels. Transcription modules showed enrichment of chromosome Y-linked genes and genes associated with mitosis in stimulated CD4, and not CD8, T-cells (Figure 5B). These findings are consistent with the gender distribution of the patients and our CD39 expression data.

Dysregulated expression of genes, including *MMP8*, in PBMCs of patients with idiopathic pulmonary fibrosis has been reported, and reduced *CD28, ICOS, LCK*, and *ITK* gene expression in PBMCs was associated with poor outcome in cohorts with this disorder (36, 37). No significant differences in gene expression of *MMP8*, 16 other matrix metalloproteases, *CD28, ICOS, LCK, ITK*, or 44 other genes of a 52-gene expression signature associated with outcome in idiopathic pulmonary fibrosis were found (Tables S2 and S3 in Supplementary Material). A list of differentially expressed genes without a log_2_-fold change threshold and FDR-adjusted *p*-values less than 0.1 in at least for one comparison is shown in Table S4 in Supplementary Material.

## Discussion

Alveolar inflammation with dysregulation of immune cells and pro-inflammatory proteins is a feature of fibrotic lung disease, but the extent of these processes beyond the lung and their role in the pathogenesis of disease are incompletely understood. A strategic approach to studying complex disorders is to use inherited diseases as models; analyses of samples from patients with FPF or HPS pulmonary fibrosis provide insights into fibrotic lung disease. Studying these oligogenic disorders with highly penetrant pulmonary fibrosis limits variability in findings related to genetic heterogeneity between patients. To expand the understanding of the immune system in pulmonary fibrosis, we performed comprehensive analyses of the blood immunome and immune cells in cohorts with HPS pulmonary fibrosis, FPF associated with telomere disease, and their URels.

Several complete blood count measurements were significantly different among the groups, and segregation of patient subsets was observed on PCA. Our findings of low WBC, polymorphonuclear leukocyte, monocyte, RBC counts, and platelet concentrations in patients with FPF may be secondary to their underlying telomere disease, because telomerase dysfunction is known to be associated with a broad spectrum of hematologic effects with varying severity (38). HPS-1 is generally associated with normal leukocyte concentrations, hemoglobin, and platelet counts (14), and significant differences in complete blood count parameters were not found between patients with HPS pulmonary fibrosis and URels. Thus, it is interesting that PCA demonstrated segregation of these two groups.

Principal component analysis of PBMC flow cytometry data did not reveal clustering of patient groups, which is consistent with our findings of no significant differences in prominent T-cell subsets or B-cells. However, concentrations of T-cell and B-cell subpopulations were significantly different. Activated central memory helper cells were upregulated in FPF compared with HPS pulmonary fibrosis. Consistent with these data, peripheral blood concentrations of IL.4 and IFN.g were significantly reduced in FPF compared with HPS pulmonary fibrosis, and several genes associated with mitosis, cell cycle control, and lymphocyte activation were upregulated in PBMCs from patients with FPF. These findings are consistent with our previous data showing alveolar lymphocytosis with activated HLA-DR^+^ CD4^+^ cells in the lungs of patients with preclinical FPF (6). In addition, patients with progressive idiopathic pulmonary fibrosis have longitudinal over expression of genes involved with host defense response, which implies that an altered alveolar microbiome in fibrotic lung disease may provide a prolonged immunologic stimulus for chronic lung injury (39). Taken together, these results demonstrate that CD4^+^ T-cell activation in the blood and bronchoalveolar space is a feature of patients with FPF, and they provide suggestive evidence that the lung is a site of antigen exposure in pulmonary fibrosis.

Our results also show high concentrations of peripheral blood B-lymphocyte populations in patients with pulmonary fibrosis. Concentrations of CD38^+^ memory CD27^−^ B-cells, IgA^+^ memory CD27^+^ B-cells, and IgM^+^ and IgD^+^ B-cells were significantly higher in patients affected with either FPF or HPS pulmonary fibrosis compared with unaffected controls. Notably, B-cell abnormalities have been identified in other fibrotic lung disorders. In patients with idiopathic pulmonary fibrosis, lung tissue specimens are reported to contain focal aggregates of B-cells, which likely originate from extra-pulmonary sites (40). Peripheral blood B-cells in idiopathic pulmonary fibrosis are more antigen-differentiated with higher plasmablast concentrations compared with normal controls, and B-cell differentiation correlates with severity of lung disease (40). Alveolar interstitial B-cell infiltration also occurs in scleroderma, an autoimmune connective tissue disease associated with pulmonary fibrosis (41).

Our findings of high peripheral blood concentrations of activated T-cell and B-cell subpopulations and leptin in our cohort with pulmonary fibrosis are interesting. B-cells express leptin receptors, and leptin activates peripheral blood B-cells and maintains B-cell homeostasis by inhibiting apoptosis, inducing proliferation, and prolonging survival (42, 43). Leptin has similar effects on T-cells (44). These results suggest that the peripheral blood milieu in FPF and HPS pulmonary fibrosis may promote T-cell and B-cell activation and population expansion. However, additional studies investigating the role of leptin are indicated to elucidate its role in regulating T-cells and B-cells in these disorders. Overall, these data provide further evidence that these immune cells may contribute to the pathogenesis of pulmonary fibrosis, perhaps *via* cytotoxic effects or secretion of pro-inflammatory or pro-fibrotic mediators.

One limitation of the study is the older age of the subjects affected with FPF compared with patients with HPS pulmonary fibrosis or controls. Thus, it is possible that the significant differences observed between patients with FPF and patients with HPS pulmonary fibrosis or URels may be due to age rather than the underlying disease. Notably, these disorders are rare, which restricts the availability of patients and research samples. Due to the limited number of samples in this study, these conclusions are suggestive in nature, and further analyses are indicated to confirm these results.

In conclusion, we identified high concentrations of activated T-cell and B-cell subpopulations associated with altered leptin and cytokine levels in peripheral blood from cohorts with FPF or HPS pulmonary fibrosis. Several genes involved with mitosis and cell cycle regulation were highly expressed and enriched in the blood immunome of patients with FPF. These results show that lymphocyte dysregulation is an extra-pulmonary phenotype of these inherited forms of pulmonary fibrosis. Further studies are indicated to elucidate the role of pulmonary and circulating immune cells in the pathogenesis of pulmonary fibrosis.

## Ethics Statement

Written informed consent in accordance with the Declaration of Helsinki was obtained from all subjects, who enrolled in protocols 09-H-0201 (Clinical Trials number, NCT00968084; Screening Protocol for Subjects Being Evaluated for Center for Human Immunology, Autoimmunity, and Inflammatory Diseases (CHI) Protocols), 10-H-0162 (Clinical Trials number, NCT01200823; Collection of Blood, Bone Marrow, Leukapheresis, and Tissue Biopsy Samples from Patients and their Family members for Center for Human Immunology, Autoimmunity, and Inflammatory Diseases (CHI) Laboratory Research Studies), 95-HG-0193 (Clinical Trials number, NCT00001456; Clinical and Basic Investigations into Hermansky–Pudlak Syndrome), and/or 04-HG-0211 (Clinical Trials number, NCT00084305; Procurement and Analysis of Specimens from Individuals with Pulmonary Fibrosis), which were approved by the Institutional Review Board of the National Heart, Lung and Blood Institute or the National Human Genome Research Institute.

## Author Contributions

SE-C, FC, YK, KO, WG, JC, SP, AB, and BG contributed to the conception or design of the work; or the acquisition, analysis, or interpretation of data for the work. All the authors drafted the work or revised it critically for important intellectual content, approved the final version to be published, and agreed to be accountable for all aspects of the work.

## Conflict of Interest Statement

The authors declare that the research was conducted in the absence of any commercial or financial relationships that could be construed as a potential conflict of interest.

## Abbreviations

BLOC, biogenesis of lysosome-related organelles complex; HPS, Hermansky–Pudlak syndrome; PBMC, peripheral blood mononuclear cell.
