# Supplementary material for: The Immunome in Two Inherited Forms of Pulmonary Fibrosis
Source: Front Immunol. 2018 Jan 31;9:76. doi: 10.3389/fimmu.2018.00076 (PMC5797737; doi:10.3389/fimmu.2018.00076)
Supplement: Supplementary file 1 [file Table_1.docx]

**Supplemental Table 1. List of analytes and kits**

| **Cytokine I** | **Cytokine II** | **Diabetes** | **Acute Phase** | **Acute Phase** | **Th17** |
| --- | --- | --- | --- | --- | --- |
| PDGF-bb | CTACK | C-peptide | PCT | A2M | IL-17A |
| IL-1b | GROa | Ghrelin | Ferritin | Haptoglobin | IL-17F |
| IL-1ra | IL-1a | GIP | tPA | CRP | IL-21 |
| IL-2 | IL-2Ra | GLP-1 | Fibrinogen | SAP | IL-22 |
| IL-4 | IL-3 | Glucagon | SAA |  | IL-23 |
| IL-5 | IL-12p40 | Insulin |  |  | IL-25 |
| IL-6 | IL-16 | Leptin |  |  | IL-31 |
| IL-7 | IL-18 | PAI-1 |  |  | IL-33 |
| IL-8 | LIF | Resistin |  |  | sCD40L |
| IL-9 | MCP-3 | Visfatin |  |  |  |
| IL-10 | M-CSF |  |  |  |  |
| IL-12 (p70) | MIF |  |  |  |  |
| IL-13 | MIG |  |  |  |  |
| IL-15 | b-NGF |  |  |  |  |
| IL-17A | SCF |  |  |  |  |
| Eotaxin | SCGF-b |  |  |  |  |
| FGF basic | SDF-1a |  |  |  |  |
| G-CSF | TNF-b |  |  |  |  |
| GM-CSF | TRAIL |  |  |  |  |
| IFN-g | HGF |  |  |  |  |
| IP-10 | IFN-a2 |  |  |  |  |
| MCP-1 (MCAF) |  |  |  |  |  |
| MIP-1a |  |  |  |  |  |
| MIP-1b |  |  |  |  |  |
| RANTES |  |  |  |  |  |
| TNF-a |  |  |  |  |  |
| VEGF |  |  |  |  |  |

All analytes were detectable in at least 1 patient, except GM-CSF, IL-3, and LIF.
