# Supplementary material for: The Immunome in Two Inherited Forms of Pulmonary Fibrosis
Source: Front Immunol. 2018 Jan 31;9:76. doi: 10.3389/fimmu.2018.00076 (PMC5797737; doi:10.3389/fimmu.2018.00076)
Supplement: Supplementary file 2 [file Table_2.docx]

**Supplemental Table 2. Expression of matrix metalloprotease genes in PBMCs**

| Gene | FPF-HPSPF (FC) | FPF-HPSPF (FDR) | FPF-UREL (FC) | FPF-UREL (FDR) | HPSPF-UREL (FC) | HPSPF-UREL (FDR) |
| --- | --- | --- | --- | --- | --- | --- |
| MMP1 | 0.12 | 0.841 | 0.03 | 0.964 | -0.08 | 0.833 |
| MMP3 | 0.08 | 0.854 | 0.03 | 0.957 | -0.05 | 0.866 |
| MMP7 | 0.11 | 0.811 | -0.07 | 0.904 | -0.18 | 0.723 |
| MMP8 | 1.07 | 0.854 | 2.14 | 0.809 | 1.06 | 0.792 |
| MMP9 | 0.71 | 0.830 | 0.4 | 0.924 | -0.31 | 0.896 |
| MMP10 | 0.12 | 0.820 | 0.05 | 0.946 | -0.07 | 0.846 |
| MMP11 | 0.05 | 0.931 | 0.03 | 0.957 | -0.02 | 0.966 |
| MMP12 | 0.04 | 0.967 | 0.03 | 0.964 | 0 | 0.996 |
| MMP13 | -0.07 | 0.898 | -0.03 | 0.959 | 0.04 | 0.916 |
| MMP14 | 0.16 | 0.540 | 0.07 | 0.893 | -0.09 | 0.778 |
| MMP16 | -0.02 | 0.975 | 0.04 | 0.946 | 0.06 | 0.849 |
| MMP17 | 0.11 | 0.911 | 0.19 | 0.865 | 0.08 | 0.899 |
| MMP19 | 0.1 | 0.899 | 0.07 | 0.937 | -0.03 | 0.959 |
| MMP20 | 0.09 | 0.838 | 0.1 | 0.865 | 0.02 | 0.965 |
| MMP25 | 0.14 | 0.916 | -0.19 | 0.895 | -0.33 | 0.744 |
| MMP26 | 0.03 | 0.972 | -0.05 | 0.957 | -0.08 | 0.864 |
| MMP28 | -0.02 | 0.971 | -0.15 | 0.712 | -0.13 | 0.723 |

FC, fold change

FDR, false-discovery rate-adjusted p-value

MMP, matrix metalloprotease
