# Supplementary material for: The Immunome in Two Inherited Forms of Pulmonary Fibrosis
Source: Front Immunol. 2018 Jan 31;9:76. doi: 10.3389/fimmu.2018.00076 (PMC5797737; doi:10.3389/fimmu.2018.00076)
Supplement: Supplementary file 3 [file Table_3.docx]

**Supplemental Table 3. Expression of 48 of 52 genes in IPF PBMC gene expression signature**

| Gene | FPF-HPSPF (FC) | FPF-HPSPF (FDR) | FPF-UREL (FC) | FPF-UREL (FDR) | HPSPF-UREL (FC) | HPSPF-UREL (FDR) |
| --- | --- | --- | --- | --- | --- | --- |
| ARHGAP5 | -0.16 | 0.841 | -0.17 | 0.868 | -0.01 | 0.996 |
| ARL4C | 0.04 | 0.984 | -0.12 | 0.939 | -0.15 | 0.857 |
| BIRC3 | -0.12 | 0.941 | 0.12 | 0.940 | 0.25 | 0.787 |
| BTN3A1 | -0.14 | 0.890 | -0.05 | 0.961 | 0.09 | 0.894 |
| BTN3A2 | -0.07 | 0.964 | 0.17 | 0.903 | 0.25 | 0.769 |
| BTN3A3 | 0.08 | 0.962 | -0.02 | 0.991 | -0.1 | 0.917 |
| C19orf59 | 0.23 | 0.867 | 0.2 | 0.902 | -0.03 | 0.983 |
| C7orf58 | -0.17 | 0.898 | -0.15 | 0.915 | 0.02 | 0.988 |
| CAMK2D | -0.13 | 0.853 | -0.04 | 0.959 | 0.09 | 0.852 |
| CD2 | -0.01 | 0.995 | 0.04 | 0.981 | 0.05 | 0.961 |
| CD28 | 0 | 0.999 | -0.11 | 0.957 | -0.11 | 0.933 |
| CD47 | -0.14 | 0.820 | -0.12 | 0.879 | 0.02 | 0.974 |
| CD96 | -0.13 | 0.941 | -0.25 | 0.888 | -0.11 | 0.918 |
| CNOT6L | -0.16 | 0.829 | -0.11 | 0.904 | 0.05 | 0.928 |
| CXCR6 | -0.09 | 0.902 | -0.06 | 0.939 | 0.03 | 0.958 |
| DOCK10 | -0.1 | 0.880 | -0.1 | 0.891 | 0 | 0.999 |
| ETS1 | -0.02 | 0.986 | -0.05 | 0.965 | -0.03 | 0.974 |
| FLT3 | 0.32 | 0.890 | 0.23 | 0.928 | -0.09 | 0.959 |
| GBP4 | -0.01 | 0.998 | 0.33 | 0.837 | 0.34 | 0.723 |
| GPR174 | -0.13 | 0.952 | -0.14 | 0.944 | -0.01 | 0.998 |
| GPR183 | 0.09 | 0.961 | -0.03 | 0.984 | -0.12 | 0.896 |
| HLA-DPA1 | 0.08 | 0.961 | -0.11 | 0.941 | -0.18 | 0.817 |
| HLA-DPB1 | -0.03 | 0.971 | -0.01 | 0.994 | 0.02 | 0.964 |
| HP | 0.37 | 0.928 | 0.89 | 0.860 | 0.52 | 0.832 |
| ICOS | 0.22 | 0.897 | 0.22 | 0.904 | 0 | 0.999 |
| IL1R2 | 0.7 | 0.820 | 0.43 | 0.906 | -0.27 | 0.909 |
| IL7R | -0.23 | 0.868 | -0.3 | 0.865 | -0.07 | 0.951 |
| ITK | 0 | 1.000 | -0.12 | 0.931 | -0.12 | 0.878 |
| KLF12 | -0.19 | 0.890 | -0.27 | 0.865 | -0.07 | 0.940 |
| LARP4 | -0.05 | 0.952 | 0.11 | 0.886 | 0.17 | 0.744 |
| LBH | -0.18 | 0.923 | -0.14 | 0.948 | 0.05 | 0.974 |
| LCK | -0.01 | 0.994 | -0.07 | 0.960 | -0.06 | 0.955 |
| LPAR6 | 0.01 | 0.993 | 0.25 | 0.809 | 0.24 | 0.723 |
| LRRC39 | 0.19 | 0.868 | -0.12 | 0.929 | -0.31 | 0.744 |
| MORC4 | -0.08 | 0.970 | -0.04 | 0.982 | 0.04 | 0.979 |
| NAP1L2 | 0.11 | 0.935 | 0.07 | 0.959 | -0.04 | 0.966 |
| NUP43 | 0.02 | 0.984 | 0.21 | 0.847 | 0.18 | 0.755 |
| P2RY10 | -0.14 | 0.959 | -0.08 | 0.970 | 0.06 | 0.970 |
| PLBD1 | -0.04 | 0.984 | 0.05 | 0.976 | 0.1 | 0.934 |
| S100A12 | 0.01 | 0.996 | 0.04 | 0.968 | 0.04 | 0.964 |
| S1PR1 | -0.05 | 0.984 | 0.02 | 0.994 | 0.07 | 0.959 |
| SH2D1A | -0.23 | 0.864 | -0.4 | 0.841 | -0.17 | 0.857 |
| SLAMF7 | -0.37 | 0.812 | 0 | 0.998 | 0.37 | 0.756 |
| STAT4 | -0.18 | 0.914 | -0.21 | 0.902 | -0.04 | 0.975 |
| TC2N | -0.19 | 0.902 | -0.21 | 0.903 | -0.02 | 0.993 |
| TPST1 | 0.33 | 0.846 | 0.42 | 0.862 | 0.09 | 0.952 |
| TRAT1 | 0.06 | 0.968 | 0.06 | 0.960 | 0.01 | 0.997 |
| UTRN | -0.17 | 0.783 | -0.22 | 0.837 | -0.05 | 0.930 |

FC, fold change

FDR, false-discovery rate-adjusted p-value
