# Supplementary material for: The Immunome in Two Inherited Forms of Pulmonary Fibrosis
Source: Front Immunol. 2018 Jan 31;9:76. doi: 10.3389/fimmu.2018.00076 (PMC5797737; doi:10.3389/fimmu.2018.00076)
Supplement: Supplementary file 4 [file Table_4.docx]

**Supplemental Table 4. Differentially expressed genes**

| Gene | FPF-HPSPF (FC) | FPF-HPSPF (FDR) | FPF-UREL (FC) | FPF-UREL (FDR) | HPSPF-UREL (FC) | HPSPF-UREL (FDR) |
| --- | --- | --- | --- | --- | --- | --- |
| APOBEC3B | 0.33 | 0.439 | 0.62 | 0.076 | 0.29 | 0.723 |
| BAX | 0.24 | 0.083 | 0.17 | 0.631 | -0.07 | 0.778 |
| BUB1 | 0.49 | 0.083 | 0.63 | 0.064 | 0.14 | 0.789 |
| BUB1B | 0.42 | 0.089 | 0.56 | 0.064 | 0.14 | 0.778 |
| C15orf42 | 0.31 | 0.098 | 0.44 | 0.053 | 0.13 | 0.744 |
| C3orf59 | 0.56 | 0.140 | 0.79 | 0.069 | 0.24 | 0.756 |
| CCNA2 | 0.49 | 0.189 | 0.75 | 0.064 | 0.26 | 0.735 |
| CCNB2 | 0.79 | 0.015 | 0.99 | 0.023 | 0.2 | 0.762 |
| DDB2 | 0.34 | 0.088 | 0.28 | 0.535 | -0.06 | 0.874 |
| DLGAP5 | 0.55 | 0.201 | 0.78 | 0.086 | 0.23 | 0.759 |
| DTL | 0.73 | 0.140 | 0.99 | 0.076 | 0.27 | 0.770 |
| EDA2R | 0.36 | 0.083 | 0.23 | 0.712 | -0.12 | 0.756 |
| FANCI | 0.33 | 0.089 | 0.35 | 0.200 | 0.02 | 0.960 |
| GINS2 | 0.34 | 0.083 | 0.44 | 0.053 | 0.1 | 0.778 |
| HIST1H1B | 0.62 | 0.089 | 0.76 | 0.083 | 0.14 | 0.841 |
| HIST1H3B | 0.83 | 0.103 | 1.15 | 0.064 | 0.32 | 0.756 |
| HPS1 | 0.38 | 0.000 | 0.08 | 0.878 | -0.3 | 0.233 |
| KIAA0101 | 0.87 | 0.089 | 1.09 | 0.076 | 0.22 | 0.825 |
| KIF11 | 0.75 | 0.083 | 0.93 | 0.069 | 0.17 | 0.830 |
| KIF20A | 0.41 | 0.083 | 0.53 | 0.053 | 0.11 | 0.787 |
| NCAPG | 0.49 | 0.089 | 0.61 | 0.076 | 0.12 | 0.825 |
| NCAPH | 0.38 | 0.089 | 0.47 | 0.076 | 0.09 | 0.832 |
| NRCAM | -0.46 | 0.423 | -1.04 | 0.038 | -0.57 | 0.723 |
| NUSAP1 | 0.47 | 0.251 | 0.74 | 0.076 | 0.27 | 0.739 |
| OR51B6 | -0.08 | 0.838 | -0.3 | 0.076 | -0.22 | 0.655 |
| PBK | 0.32 | 0.083 | 0.4 | 0.064 | 0.08 | 0.809 |
| PLK1 | 0.47 | 0.083 | 0.72 | 0.023 | 0.25 | 0.723 |
| PRC1 | 0.38 | 0.089 | 0.4 | 0.263 | 0.02 | 0.976 |
| PTGER2 | 0.28 | 0.140 | 0.41 | 0.064 | 0.14 | 0.744 |
| SOCS2 | 0.39 | 0.351 | 0.66 | 0.076 | 0.27 | 0.729 |
| TPX2 | 0.52 | 0.103 | 0.78 | 0.053 | 0.26 | 0.735 |
| TRIP13 | 0.21 | 0.089 | 0.34 | 0.030 | 0.13 | 0.723 |
| TYMS | 0.68 | 0.083 | 0.85 | 0.067 | 0.17 | 0.823 |

FC, fold change

FDR, false-discovery rate-adjusted p-value
